# Three-dimensional spherical spatial boundary conditions differentially regulate osteogenic differentiation of mesenchymal stromal cells

**DOI:** 10.1038/srep21253

**Published:** 2016-02-17

**Authors:** Yin-Ping Lo, Yi-Shiuan Liu, Marilyn G. Rimando, Jennifer Hui-Chun Ho, Keng-hui Lin, Oscar K. Lee

**Affiliations:** 1 Program in Molecular Medicine, National Yang-Ming University and Academia Sinica, Taipei 11221, Taiwan; 2 Stem Cell Research Center, National Yang-Ming University, Taipei 11221, Taiwan; 3 Taiwan International Graduate Program in Molecular Medicine, National Yang-Ming University and Academia Sinica, Taipei 11221, Taiwan; 4 Center for Stem Cell Research, Wan Fang Hospital, Taipei Medical University, Taipei 11696, Taiwan; 5 Graduate Institute of Clinical Medicine, Taipei Medical University, Taipei 11031, Taiwan; 6 Department of Ophthalmology, Wan Fang Hospital, Taipei Medical University, Taipei 11696, Taiwan; 7 Institute of Physics, Academia Sinica, Taipei 11529, Taiwan; 8 Taipei City Hospital, Taipei 10341, Taiwan; 9 Institute of Clinical Medicine, National Yang-Ming University, Taipei 11221, Taiwan; 10 Department of Orthopaedics and Traumatology, Taipei Veterans General Hospital, Taipei 11217, Taiwan

## Abstract

The spatial boundary condition (SBC) arising from the surrounding microenvironment imposes specific geometry and spatial constraints that affect organogenesis and tissue homeostasis. Mesenchymal stromal cells (MSCs) sensitively respond to alterations of mechanical cues generated from the SBC. However, mechanical cues provided by a three-dimensional (3D) environment are deprived in a reductionist 2D culture system. This study investigates how SBC affects osteogenic differentiation of MSCs using 3D scaffolds with monodispersed pores and homogenous spherical geometries. MSCs cultured under SBCs with diameters of 100 and 150 μm possessed the greatest capability of osteogenic differentiation. This phenomenon was strongly correlated with MSC morphology, organization of actin cytoskeleton, and distribution of focal adhesion involving α2 and α5 integrins. Further silencing either α2 or α5 integrin significantly reduced the above mentioned mechanosensitivity, indicating that the α2 and α5 integrins as mechano-sensitive molecules mediate MSCs’ ability to provide enhanced osteogenic differentiation in response to different spherical SBCs. Taken together, the findings provide new insights regarding how MSCs respond to mechanical cues from the surrounding microenvironment in a spherical SBC, and such biophysical stimuli should be taken into consideration in tissue engineering and regenerative medicine in conjunction with biochemical cues.

Stem cells within organs or tissues constantly probe and actively respond to a variety of mechanical stimuli from their surrounding microenvironment. In addition to biochemical cues, mechanical cues have been shown to play critical roles in organogenesis and tissue homeostasis^1^,^2^. On the other hand, organ or tissue architectures serve as structure-based scaffolding and provide a source of natural mechanical cues for cells. At the single cell level, the spatial boundary condition (SBC) determined by the spatial presentation of extracellular matrix (ECM) and surrounding cells imposes a unique structural geometry and spatial constraint that affects stem cell self-renewal and differentiation, specifically in mesenchymal^3^, hematopoietic^4^, cardiac^5^, keratinocytic^6^, and hair follicle stem cells^7^. Application of mechanical stimuli to manipulate cell behavior offers several advantages. For example, mechanical forces can be directionally summed, thus amplifying the net effect of mechanotransduction by increasing the magnitude of the optimal force applied^8^. For this reason, the mechanical properties of microenvironments have been explored as another regulatory factor to precisely control stem cell fate and function *in situ*.

Mesenchymal stromal cell (MSC)-based therapies have great potential in regenerative medicine as MSCs not only can be isolated easily and propagated *in vitro* but also are multipotent cells with low immunogenicity^9^,^10^. Accumulated evidence has indicated the therapeutic value of MSCs in rebuilding damaged or diseased tissue, especially for bone and cartilage repair^11^, cardiac regeneration^12^, and treatment of neurodegenerative disorders^13^. Manipulation of the mechanical parameters of a two-dimensional (2D) substrate by modulating adhesive material elasticity^14^,^15^, ligand patterning^16^,^17^,^18^, or substrate topography^19^,^20^ has been reported to affect MSC proliferation, differentiation, migration, and apoptosis. The regulatory mechanism of mechanical properties on stem cell behaviors is mainly based on results obtained from 2D flat systems and thus may not represent the exact stem cell behaviors in three-dimensional (3D) scenarios. Better understanding the mechanism of natural 3D milieu governing biological characteristics and functions of MSCs is needed for effective clinical applications.

MSCs are located in trabecular bone consisting of various 3D microporous pores with porosity ranging from 50–90%^21^. The unique porous structure determines the mechanical properties of trabecular bone as impairment of the bone microarchitecture is associated with an increased risk of fracture^22^. Previous studies related to bone microarchitecture have demonstrated that gelatin spongy^23^ induces osteoblast differentiation of MSCs, and implantation of trabecular bone microarchitecture-based biphasic calcium phosphate ceramic scaffolds with MSCs can repair the load-bearing bone defect *in vivo*^24^. In general, macropore or high interconnected porosity not only is essential to provide space for vascularization and tissue ingrowth but also facilitates mass transport. On the other hand, a denser structure or low interconnectivity increases the mechanical stiffness of the scaffold^25^. The bionic materials used in the above mentioned studies have a wide range of pore sizes, irregular spatial boundaries, and various porosities and have thus limited the investigation of how SBC influences stem cell behaviors.

In this study, the SBC provided by 3D scaffold with monodispersed pores and homogenous spherical geometries is precisely controlled. 3D scaffolds with the same porosity but different SBCs (i.e. pore diameters) are used to investigate how spherical SBCs affect the osteogenic differentiation of MSCs. We hypothesize that the spherical SBCs differentially regulate osteogenic differentiation of MSCs through mechanical sensing and cytoskeleton remodeling.

## Results

### 3D structural geometries and characteristics of the fabricated scaffolds

**Figure 1:**
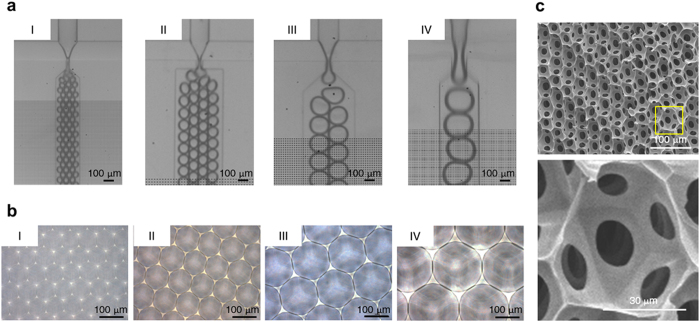
Fabrication of the 3D scaffolds with homogenous spherical geometries and controllable diameters. (**a**) Micrographs of bubbles generated in a focused flow where the bubble size was controlled in the microfluidic device. (**b**) Micrographs of the fabricated scaffolds with size-defined pores. (**c**) Scanning electron micrographs of the cross-section of freeze-dried 3D scaffolds with homogenous spherical geometries and interconnected networks throughout the scaffold interior. Note that the magnified image displayed tunnel formation between pores.

Four groups with size-defined bubbles were generated using a novel microfluidic device (Fig. 1a and Supplementary Fig. S1). Figure 1b shows the resulting, highly ordered and uniform-sized micropores were imaged by bright field microscopy, showing pore diameters of 47.0 ± 2.2 μm (Group I), 84.8 ± 11.0 μm (Group II), 147.9 ± 7.2 μm (Group III), and 198.7 ± 9.1 μm (Group IV). The ultrastructural characteristics of the scaffold were examined by SEM. The fabricated scaffolds exhibited homogenous three-dimensional (3D) spherical geometries with interconnected networks throughout the scaffold interior (Fig. 1c). The above results demonstrated that our manufacturing method yielded reproducible 3D scaffolds to be used for subsequent studies.

Since the mechanical properties of the scaffold play a significant role in tissue regeneration^25^, the properties of the 3D structural geometry associated with different pore sizes were further characterized and listed in Supplementary Table S1. The ratio of surface negative curvature was respectively 12: 6: 4: 3 in Groups I, II, III and IV. A larger pore size has a smaller curvature since the curvature is reciprocal to the radius (r). We further validated the total porosity and elastic characteristic of the fabricated scaffolds. Using the gravimetric method, the porosity of the porous scaffold (Φ) was calculated and represented as Equation (1)


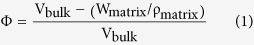


where V_bulk_ is bulk volume, W_matrix_ is total weight of the matrix, and ρ_matrix_ is mean density of 7% gelatin (1.0 mg/ml). Four scaffolds with different pore diameters were at the same porosity of 69.7 ± 2.3%. Furthermore, rheological measurement demonstrated a similar elastic characteristic of the fabricated scaffolds with storage moduli of 3.3 ± 0.6 KPa (Group I), 3.4 ± 0.8 KPa (Group II), 4.1 ± 0.5 KPa (Group III), and 3.6 ± 0.5 KPa (Group IV), indicating that alteration of pore sizes of these scaffolds did not affect the mechanical property given the same porosity.

### Cell viability of MSCs was independent of 3D spherical spatial boundary conditions (SBCs)

**Figure 2:**
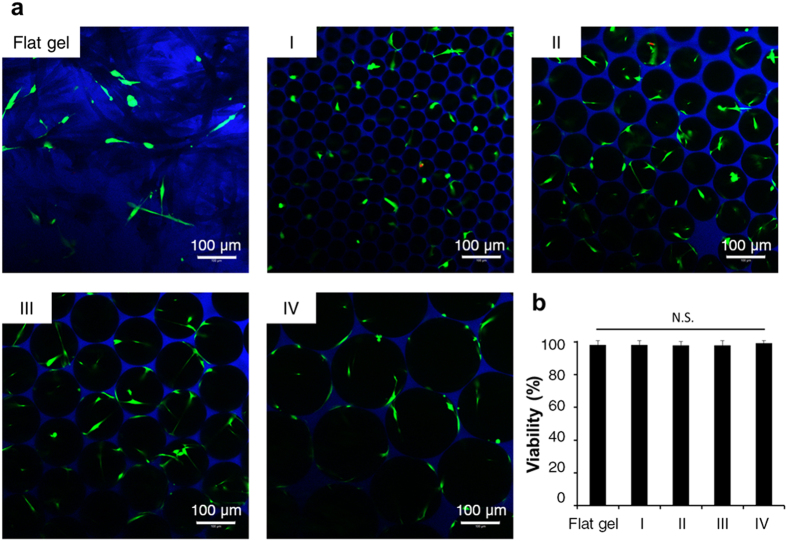
Cell viability of MSCs in the 3D scaffolds (Groups I, II, III, and IV) and on the 2D flat gel. (**a**) Fluorescence micrographs of live and dead MSCs in the 3D scaffolds or on the 2D flat gel at 1 day of culturing. Live cells were stained with calcein-AM (green) and dead cells were stained by ethidium homodimer-1 (red). (**b**) Analysis and quantification of cell viability from five random microscopic fields at 100 × magnification were displayed as the percentage of live cells to total cell numbers. Data were represented as mean ± S.D., *n* = 5. N.S., no significance.

To study whether spherical SBC affects cell survival, cell viability was analyzed by live and dead staining after 1 day of culturing and monitored by MTS assay after 1, 2, 3, 4, and 5 days of culturing in a maintenance medium. Fluorescence micrographs showed that live cells were stained green, whereas dead cells with compromised membranes were stained red (Fig. 2a). The cell survival rate in the 3D scaffolds was close to that on the 2D flat gel, which was 98.1 ± 2.6%. Among these four groups, viabilities of MSCs were 98.2 ± 2.5% (Group I), 98.1 ± 2.2% (Group II), 97.9 ± 3.0% (Group III), and 99.4 ± 1.3% (Group IV), indicating that most cells were alive and cell viability was unaffected by pore size (Fig. 2b). A similar phenomenon is shown in Supplementary Fig. S2, where the cell survival rates of MSCs in the 3D scaffolds are similar at each time point. Approximately 2- and 2.5-fold increases in cell viability were respectively detected in all study groups after 3 and 4 days of culturing. The above results demonstrated that the fabricated scaffolds were cytocompatible and sufficient for cell growth.

### Morphological changes of MSCs in response to 3D spherical SBCs

**Figure 3:**
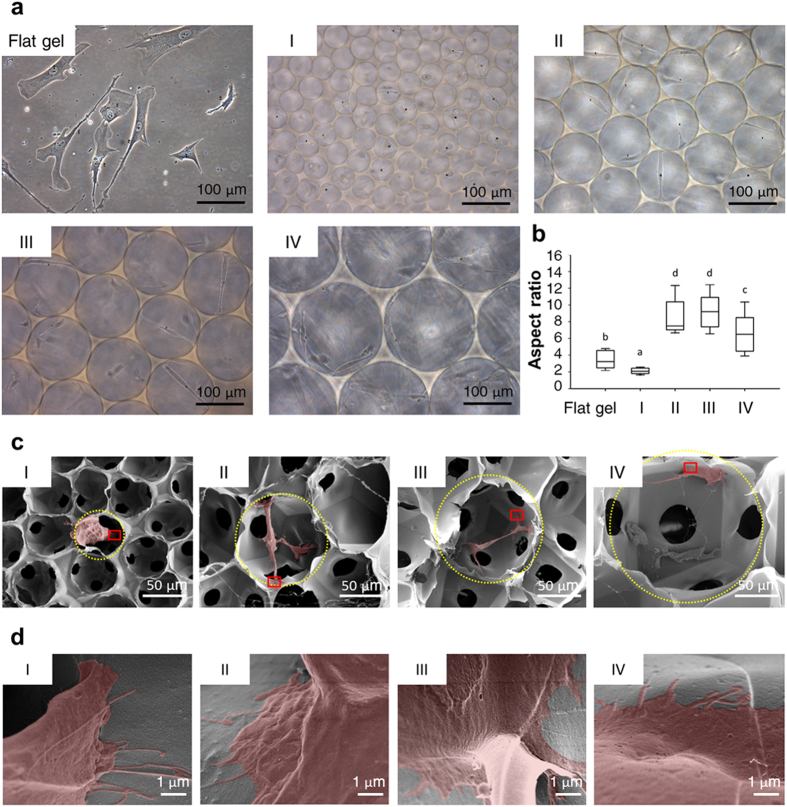
Ultrastructural analysis of MSCs morphology in the 3D scaffolds (Groups I, II, III, and IV). (**a**) Micrographs of MSC shapes in the 3D scaffolds or on the 2D flat gel at 1 day of culturing. (**b**) Aspect ratio (AR) in a cell from the z-projected image was displayed as a ratio of the long to short axes, and measured by ImageJ software. The boxplot whisker ends ranged from 5–95% and the middle line displayed the median, *n* = 20 cells. Groups with different letters were significantly different, whereas groups with same letters were not; *p* < 0.05. (**c**) Scanning electron micrographs of freeze-dried MSCs (red) in the 3D scaffolds revealed cells balancing their position by extending the protrusions to keep themselves from detaching from the matrix. The spatial boundaries were marked by the yellow dotted line. (**d**) The magnified box highlighted the interface interaction between the MSC (red) and the surrounding matrix.

The scaffolds with different SBCs (Groups I, II, III, and IV) provided unique spherical geometries and spatial constraints for MSCs (Figs 1 and 2). We next investigated the effect of SBCs on MSC morphology using bright field microscopy and SEM. Cell shapes were analyzed by ImageJ and were presented as an aspect ratio (AR). Live cell imaging showed that MSCs stretched within Groups II and III significantly increased the AR at 8.9 ± 2.7 (Group II) and 9.1 ± 2.2 (Group III) (Fig. 3a,b). In contrast, MSCs in Group IV were flattened and attached to the walls with reduced an AR value of 6.6 ± 2.3, and those grown in Group I had a nearly spherical shape with a small AR of 2.1 ± 0.4. Cells on a flat gel also had lower AR values of 3.5 ± 1.1. These findings were further supported by SEM micrographs of MSCs grown in each pore. Ultrastructural images demonstrated that MSC stretched out cell protrusions and grasped the surrounding matrix in Groups II and III. In Groups I and IV, the whole cell attached to the wall (Fig. 3c,d). These results indicated that MSCs used protrusions to balance their position in 3D spaces with different SBCs and kept themselves from detaching from the matrix.

### 3D spherical SBCs differentially modulated osteogenic differentiation of MSCs

**Figure 4:**
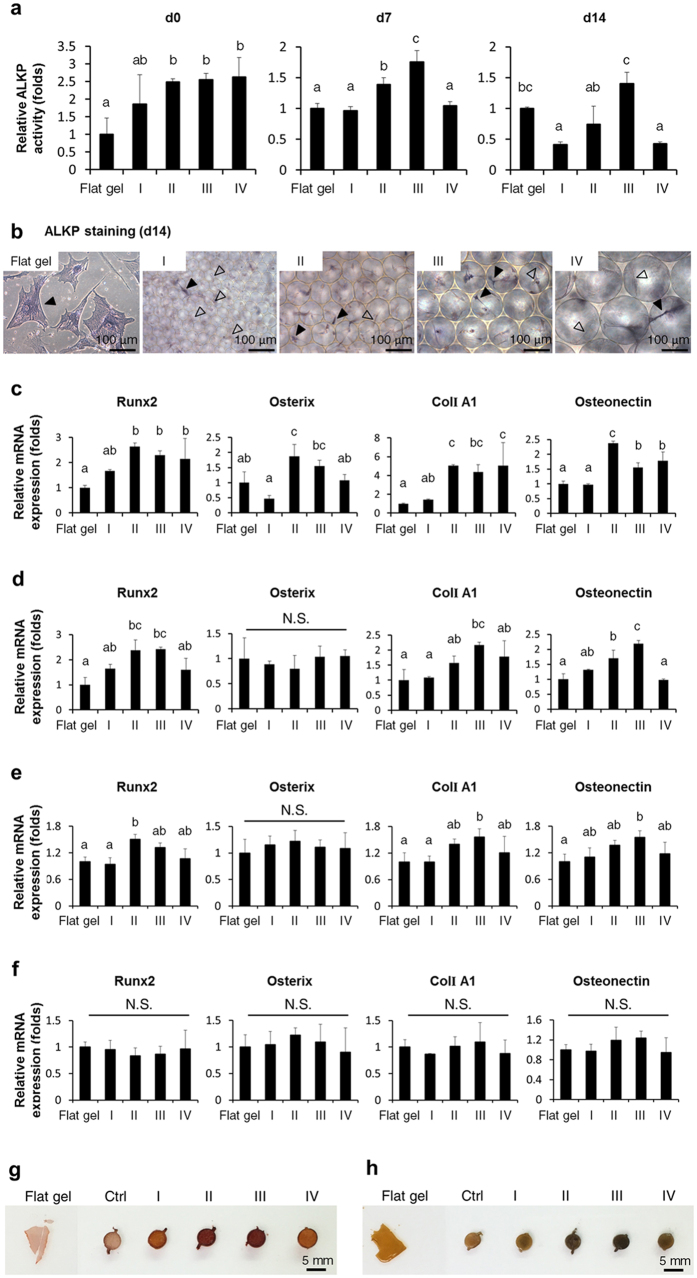
Osteogenic differentiation of MSCs in the 3D scaffolds (Groups I, II, III, and IV) and on the 2D flat gel. (**a**) Analysis and quantification of ALKP activity of differentiating MSCs in the 3D scaffolds or on the 2D flat gel at 0, 7, and 14 days of culturing in osteogenic medium. (**b**) ALKP staining of differentiating MSCs in the 3D scaffolds or on the 2D flat gel at 14 days of culturing in osteogenic medium. ALKP positive cells were stained bluish-purple. Solid arrows indicated positively stained cells and hollow arrows indicated negative staining. Osteoblast-related gene expressions of differentiating MSCs in the 3D scaffolds or on the 2D flat gel were determined by qPCR after (**c**) 1 day of culturing in the maintenance medium and (**d**) 7, (**e**) 14, and (**f**) 21 days of culturing in the osteogenic medium. Data were represented as mean ± SD of the ratios of the 3D groups to the flat gel group, *n* = 3. Groups with different letters were significantly different, whereas groups with same letters were not; *p* < 0.05. N.S., no significance. (**g**) Alizarin red S and (**h**) von Kossa staining of differentiating MSCs in the 3D scaffolds or on the 2D flat gel at 28 days of culturing in the osteogenic medium. Ctrl represented control group with 3D scaffold only. Alizarin red S colored calcium deposits were stained red. Von Kossa staining demonstrated blackened in calcium salts.

To determine the impact of spherical SBCs on the osteogenic differentiation of MSCs, the differentiation ability of MSCs was evaluated by ALKP activity, a marker used to assess osteogenic lineage commitment and differentiation^26^. ALKP activity of all groups gradually increased during differentiation after induction for 0, 7, and 14 days (see Supplementary Fig. S3). Among the study groups, the ALKP activity of Groups II and III was approximately 1.5- to 2-fold higher than that of Groups I and IV at 7 and 14 days of culturing in osteogenic medium (Fig. 4a). In contrast, ALKP activity was similar for Group I and IV. ALKP staining at 14 days demonstrated that a large majority of the cells in Groups II and III were positively stained. Intriguingly, positively stained cells stretched over and attained a balanced position within the micropores (Fig. 4b). These findings support our hypothesis that 3D spherical SBC affects osteogenic differentiation of MSCs.

The effect of spherical SBCs on the osteogenic potential of MSCs was further investigated. Transcription levels of osteoblast-related genes were determined by qPCR, including two early marker genes, transcription factors *runt-related transcription factor 2 (Runx2)* and *osterix*, and two bone matrix-related genes, *Type I collagen alpha1 (ColI A1)* and *osteonectin*^27^. The osteoblast-related genes of all study groups gradually upregulated during differentiation after induction for 0, 7, 14, and 21 days (see Supplementary Fig. S4). In the absence of osteogenic medium, expressions of all osteoblast-related genes were upregulated when MSCs were maintained in Groups II and III for 1 day (Fig. 4c). When MSCs were cultured in osteogenic medium for 7 and 14 days, higher levels of *Runx2, ColI A1,* and *osteonectin* transcripts were detected in Groups II and III, whereas no significant difference was observed between Groups I and IV (Fig. 4d,e). In contrast, *osterix* mRNA levels were not statistically different. Expressions of osteoblast-related genes were similar among all study groups at 21 days in osteogenic medium (Fig. 4f). These results indicated that spherical SBCs altered osteogenic differentiation. In particular, Groups II and III exhibited the greatest potential to accelerate osteogenic differentiation.

Furthermore, how spherical SBCs affected calcium deposition of osteogenic differentiated MSCs was investigated by alizarin red S and von Kossa staining. Positively stained alizarin red S-calcium complexes were accumulated either within the cavity or around the spatial boundary of the 3D scaffold in Groups II and III after induction of osteogenic medium for 28 days (Fig. 4g). In contrast, a small number of calcium deposits were displayed in Groups I and IV and on the flat gel group at 28 days of culturing in the osteogenic medium. These findings were further supported by the results of von Kossa staining (Fig. 4h) and clearly demonstrated that Groups II and III possessed the greatest potential to enhance osteogenic maturation. It is worth noting that the fabricated scaffold was good for trapping the minerals.

### Actin cytoskeleton reorganization and focal adhesion (FA) enhancement of MSCs in response to 3D spherical SBCs

**Figure 5:**
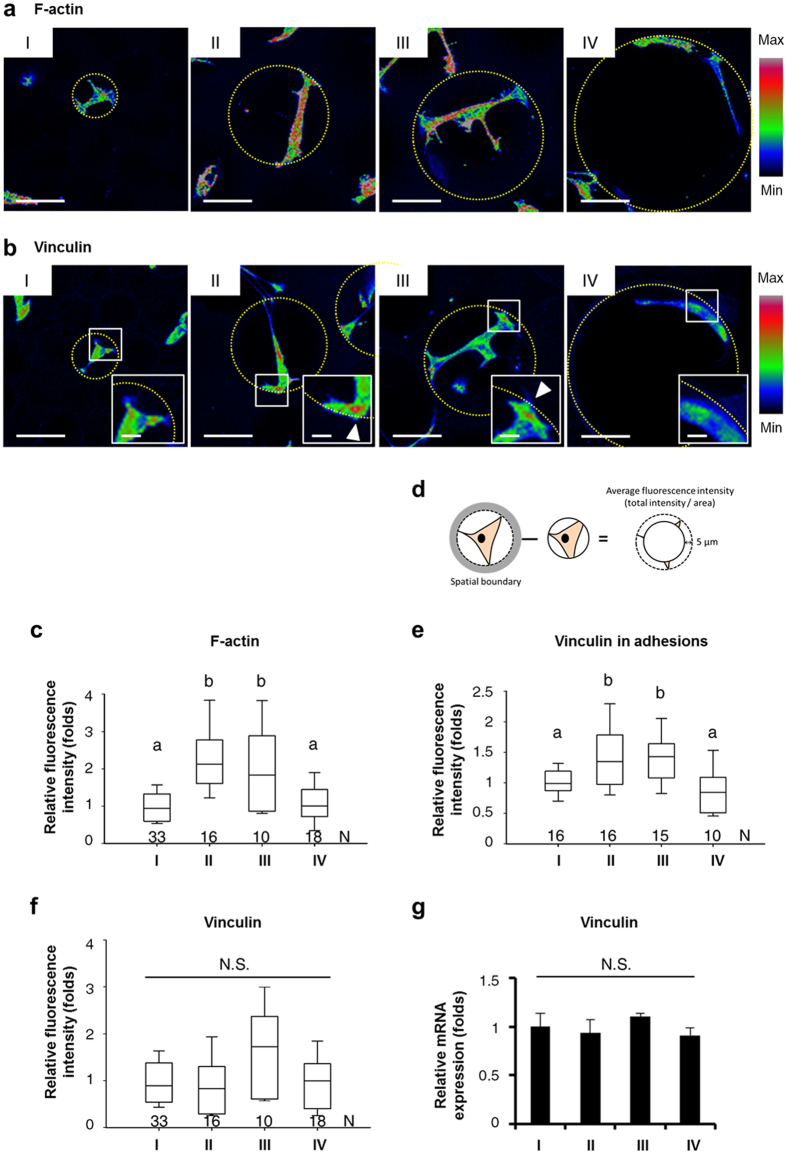
Organization and quantification of F-actin and vinculin from MSCs in the 3D scaffolds (Groups I, II, III, and IV). Fluorescent color maps of z-projection resulting from fluorescent staining demonstrated (**a**) F-actin networks and (**b**) vinculin distributions of MSCs in the 3D scaffolds. The condensation of vinculin in the adhesions was displayed in the inset and indicated by arrows. The spatial boundaries were marked by the yellow dotted line. Scale bar, 50 μm (a and b); 10 μm (b, inset). Analysis and quantification of (**c**) F-actin formation, (**e**) vinculin in adhesions, and (**f**) vinculin expressions from immunofluorescence intensities using Metamorph software. N, number of cells (**c** and **f**) or number of pores (**e**) for analysis in each group. The boxplot whisker ends ranged from 5–95% and the middle line displayed the median. (**d**) Diagram depicting the immunofluorescence intensity of vinculin in adhesions within 5 μm of the spatial boundary. The average fluorescence intensity of vinculin was measured from total intensity and normalized to cell area. (**g**) Gene expression of vinculin from MSCs in 3D scaffolds was determined by qPCR after 1 day of culturing. Data were represented as mean ± SD, *n* = 3. Fluorescence intensity and gene expression level were normalized by those of the MSCs in Group I. Groups with different letters were significantly different, whereas groups with same letters were not; *p* < 0.05. N.S., no significance.

Since morphological changes of MSCs and enhancement of interface interaction between MSCs and the surrounding matrix in response to different spherical SBCs were associated with accelerated osteogenesis (Figs 3 and 4), we next investigated the relationships between the actin cytoskeleton, FA, and osteogenic differentiation ability of MSCs under these conditions. F-actin was stained with phalloidin and observed by confocal microscopy. F-actin intensity heat maps from z-stacks showed that MSCs in Groups II and III had actin bundles of aligned long filaments. In contrast, MSCs in Groups I and IV were meshwork-like with intermingled shorter filaments and appeared as organized actin node structures (Fig. 5a). FA was studied by immunofluorescence staining of vinculin, an essential regulator of FA formation^28^, and observed by confocal microscopy. Vinculin intensity heat maps from z-stacks demonstrated that in Groups II and III vinculin abundantly clustered at the extreme ends of the cell body. In contrast, vinculin was evenly distributed throughout the cell body in Groups I and IV (Fig. 5b). Further quantification of F-actin formation from fluorescence intensity revealed a significantly greater amount of F-actin in Groups II and III than in Groups I and IV (Fig. 5c). A quantitative analysis of vinculin densities at the cell extremities by immunofluorescence intensity also agreed with the z-stack imaging (Fig. 5d,e), whereas quantification of vinculin protein levels by measuring the immunofluorescence intensity (Fig. 5f) and transcription levels by qPCR (Fig. 5g) showed that expressions of vinculin remain unaffected by spherical SBCs, suggesting that cytoplasmic vinculin tended to cluster into FA to strengthen adhesion. Additionally, the increase in both active phosphorylated myosin and FA sizes at the extreme ends of cell body was found in Groups II and III (see Supplementary Figs S5 and S6.), indicating an increase in cytoskeleton tension in Groups II and III. The above findings indicated that F-actin organization and FA distribution of MSCs were influenced by spherical SBCs and were correlated with more rapid osteogenesis (Figs 4 and 5), suggesting the role of spherical SBCs in regulating osteogenesis of MSCs was mediated by FA and actin cytoskeleton remodeling.

### 3D spherical SBCs altered the expressions of α2 and α5 integrins

**Figure 6:**
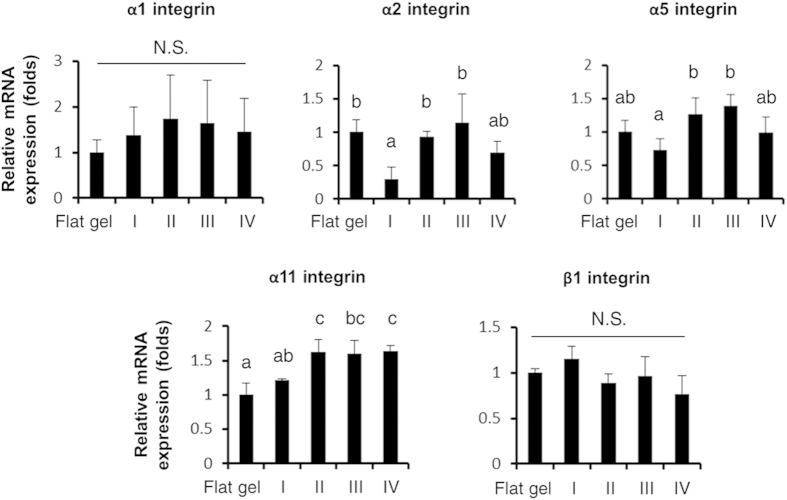
Gene expression of integrins from MSCs in the 3D scaffolds (Groups I, II, III, and IV) and on the 2D flat gel. Transcription levels of *α1, α2, α5, α11,* and *β1 integrins* of MSCs grown in the 3D scaffolds or on the 2D flat gel after 1 day of culturing were determined by qPCR. Data were represented as mean ± S.D. of the ratios of 3D groups to flat gel group, *n* = 3. Groups with different letters were significantly different, whereas groups with same letters were not; *p* < 0.05. N.S., no significance.

To further elucidate how stimulation of spherical SBCs was transduced across cell membranes into cells and affected osteogenesis, we analyzed the gene-expression profile of α1, α2, α5, α11, and β1 integrin subunits which either have a high collagen-binding affinity^29^ or are dominant in MSCs during osteogenic differentiation^30^,^31^. Gene expressions were determined by qPCR after 1 day of culturing in each study group. As shown in Fig. 6, the transcription levels of *α2 integrin* of Groups II and III were approximately 2-fold higher than those in Groups I and IV, whereas the results between Groups I and IV were not statistically different. A 1.5- to 2-fold increase in *α5 integrin* gene expressions was detected in Groups II and III compared to Groups I and IV, whereas no significant difference was observed between Groups I and IV. Furthermore, expression levels of *α11 integrin* in Groups II and III were 1.3-fold higher than those in Group I and similar to Group IV. Among each group, the *α1* and *β1 integrins* transcripts were not statistically different. These findings suggest that upregulation of *α2* and *α5 integrins* was influenced by spherical SBCs (Groups II and III) and strongly correlated with accelerated osteogenic maturation.

### 3D spherical SBCs-related acceleration of osteogenic differentiation was mediated and regulated by α2 and α5 integrins

**Figure 7:**
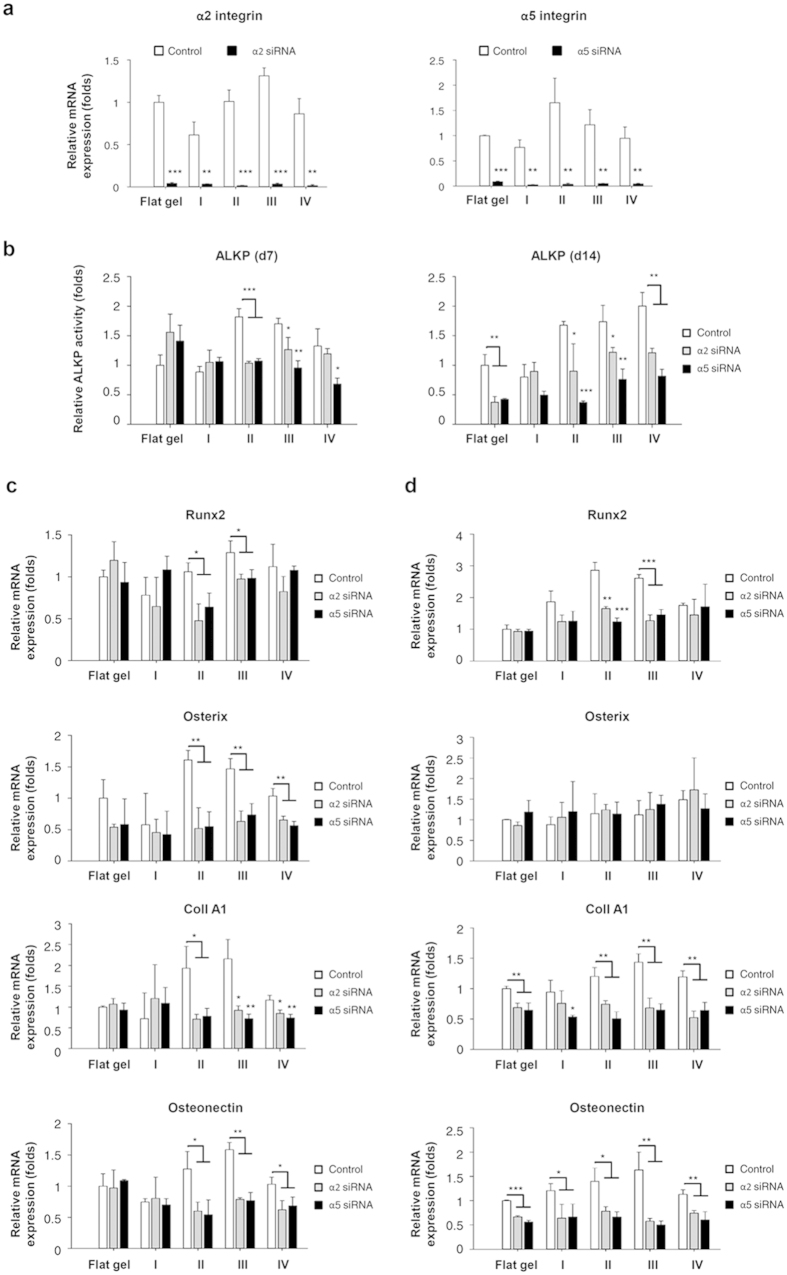
Osteogenic differentiation of MSCs in the 3D scaffolds (Groups I, II, III, and IV) and on the 2D flat gel after knockdown of α2 or α5 integrin. (**a**) Expressions of *α2* and *α5 integrins* mRNA decreased to less than 20% after siRNA knockdown for 2 days. (**b**) Comparison of ALKP activity between α2 or α5 integrin silencing and controls. ALKP activity of differentiating MSCs in the 3D scaffolds or on the 2D flat gel was analyzed at 7 and 14 days of culturing in the osteogenic medium. Comparison of transcription levels of *Runx2, osterix, ColI A1,* and *osteonectin* between α2 or α5 integrin silencing and controls. Transcription levels of differentiating MSCs in the 3D scaffolds or on the 2D flat gel were determined by qPCR at (**c**) 1 day of culturing in the maintenance medium and (**d**) 7 days in the osteogenic medium. Data were represented as mean ± SD, *n* = 3. Results were normalized by those of MSCs on the flat gel with the control group. Group with inhibition was compared to that without. Significant difference (Student’s *t*-test; *p < 0.05, **p < 0.01, ***p < 0.001) was indicated by asterisks.

Since the gene expressions of α2 and α5 integrins were upregulated in Groups II and III (Fig. 6), and integrins are the fundamental components in FA as well as the essential elements for mechanosensing and mechanotransduction^32^, we further investigated whether the spherical SBCs-mediated differentiation was regulated by α2 and α5 integrins. Downregulation of endogenous α2 or α5 integrin was induced by siRNA silencing during differentiation, and osteogenic differentiation ability was determined by quantifying ALKP activity and osteoblast-related gene expression. Results demonstrated that gene expressions of α2 or α5 integrin were reduced to less than 20% after silencing for 2 days (Fig. 7a). Silencing of α2 or α5 integrin blunted the spherical SBCs (Groups II and III)-enhanced ALKP activity at 7 days of culturing in osteogenic medium to a level similar to that found in Groups I and IV, and on flat gel groups with control. At 14 days of culture in osteogenic medium, ALKP activities were abolished in all but Group I after α2 or α5 integrin knockdown (Fig. 7b).

A comparison of osteoblast-related gene expressions between MSCs with and without siRNA knockdown further confirmed this phenomenon. Silencing α2 or α5 integrin abolished the spherical SBCs (Groups II and III)-enhanced expressions of *Runx2, osterix, ColI A1,* and *osteonectin* at 1 day of culturing in the maintenance medium to a level similar to that found in Group I and on flat gel groups with control (Fig. 7c). Furthermore, a similar phenomenon was noted in Groups II and III of α2 or α5 integrin knockdown after induction for 7 days, except the transcription levels of *osterix*. A significant decrease in *ColI A1* and *osteonectin* transcripts was also found in all study groups except for α2 integrin silenced-Group I after α2 or α5 integrin knockdown (Fig. 7d). The results demonstrated that spherical SBCs (Groups II and III)-related osteogenic differentiation was regulated by α2 and α5 integrins, suggesting that both α2 and α5 integrins were involved in the modulation of the spherical SBCs-mediated mechanically driven osteogenesis.

## Discussion

This study uses a unique method to fabricate the cytocompatible scaffolds with homogenous spherical geometries and controllable pore sizes at a micrometer scale using a novel microfluidic device (Fig. 1 and Supplementary Fig. S1). The unique scaffold system sustains adhesion, survival, and growth of mesenchymal stromal cells (MSCs) in the 3D context (Figs 2, 3, and Supplementary Fig. S2), and helps clarify the molecular mechanism underlying the effect of the spherical spatial boundary conditions (SBCs) on osteogenic differentiation of MSCs. The spherical SBCs with diameters of 100 and 150 μm are found to possess the greatest potential for osteogenic differentiation of MSCs (Fig. 4, Supplementary Figs S3, and S4). The spherical SBCs-mediated osteogenic differentiation of MSCs is strongly correlated with morphological change, organization of actin cytoskeleton, and distribution of focal adhesion (FA) involving α2 and α5 integrins (Figs 3, 4, 5, 6, Supplementary Figs S5, and S6). Of particular importance is that knockdown of either α2 or α5 integrin diminishes MSCs’ ability to enhance osteogenic differentiation in response to different spherical SBCs (Fig. 7). The unique scaffold system may serve as a useful platform for the study of cellular mechanobiology for the proliferation, migration, and differentiation of MSCs in 3D contexts. Moreover, the findings of optimal geometry and spatial constraints may offer new insights into designing next-generation 3D scaffolds for skeletal tissue engineering applications.

The SBC in 3D microenvironments has been shown to significantly influence cell architecture, cell polarity, and cell function in ways that differ from a 2D flat system. Specifically, the 3D SBC and 2D conditions differ in terms of the space available for cell spreading and migration^3^,^33^, the available extracellular matrix (ECM) for cell adhesion and contraction^34^,^35^, and the cell-cell and cell-ECM adhesion structures^36^. SBC exerts a mechanical force on cells and induces transmembrane signals through the recruitment of FA complexes^28^,^37^,^38^,^39^. In addition to bridging intracellular molecules via binding to actin-associated proteins such as vinculin and talin^28^, FA is an important hub to integrate extracellular signals via concentrating signaling transduction enzymes such as Src kinase and focal adhesion kinase (FAK)^40^. Activation of FAK or Src signaling plays an important role in regulating chemical-stimulated or ECM-induced osteogenic differentiation of MSCs^41^,^42^. Recent studies have demonstrated that, in living cells recruitment of vinculin to FA is necessary to stabilize FA formations^40^. Consistent with this, the present study finds that the specific SBC controls the spatial distribution of FA. An increase in vinculin clustering at adhesion sites is observed in spherical SBCs with diameters of 100 and 150 μm where the protrusions increase, thus extending the interface between MSCs and the surrounding matrix (Figs 3, 5, and Supplementary Fig. S5). Such phenomena are correlated with the accelerated osteogenesis of MSCs (Fig. 4). It is likely that the influence of the spherical SBCs in regulating differentiation of MSCs are transmitted and converted into biochemical signals through actively modulating FA-mediated signaling pathways.

An increase in cellular contractile force promotes osteogenesis of MSCs through actomyosin-mediated signaling pathway^17^,^32^,^33^. The contractile force that actin exerts on a single FA is proportional to the FA size^43^. The magnitude of contractile force is regulated by cell morphology^44^. Previous studies using elastic micropatterned substrate^45^ and flexible microneedle^44^ have calculated that the force exerted at a single FA is about 4–5 nN/μm^2^. We consistently detect the increase in both fluorescent densities of p-myosin and FA sizes at the extreme ends of cell body in the Groups II and III (Figs 3, 5, Supplementary Figs S5, and S6). It is likely that the spherical SBC potentially controls the maximum extension allowed for an MSC and results in FA reconfiguration as well as adhesion strengthening. Meanwhile, a force balance between intracellular cytoskeleton contractile stresses and the resistant forces generated from the spherical SBC is achieved. In support of this concept, the maximum extension is demonstrated from the differentiating positively ALKP stained MSCs (Figs 3 and 4). Thus, an alternative mechanism of the spherical SBC-mediated osteogenesis may involve cellular contractility-related signaling pathways. Further studies are needed of the spatial and temporal changes in cellular contractile force in response to different spherical SBCs to provide a clearer understanding of biophysically induced cell differentiation.

The integrin family is the fundamental components of FA^29^. It is crucial to cell survival, migration, and differentiation of MSCs by functioning as cell adhesion mediators to ECM^46^. The α2 integrin is a major receptor for collagen type I and controls collagen synthesis in osteoblasts^47^. Our previous study demonstrates its role in mechanosensitivity of the 2D matrix stiffness^48^ as well as the length of silicon nanowire^19^ during osteogenesis of MSCs. In addition to α2 integrin, the activation of α5 integrin, a cell surface receptor for fibronectin, is required and sufficient for osteoblast differentiation of MSCs under dexamethasone-induced conditions^31^. Switching between relaxed and tensioned states of α5β1 integrin activates signals through FAK in response to myosin II-generated cytoskeleton force^49^. In the present study, upregulation of *α2* and *α5 integrins* was detected when MSCs were cultured in Groups II and III (Fig. 6). Further silencing either α2 or α5 integrin abolished the spherical SBCs (Groups II and III)-enhanced expressions of *Runx2, osterix, ColI A1*, and *osteonectin* without the induction of osteogenic medium (Fig. 7c). Our results strongly suggest that α2 and α5 integrins are required to mediate or adapt different spherical SBCs and they function as mechano-sensitive molecules in the mechanosensing machinery.

Integrins are heterodimers containing an α and a β subunit. The α subunit is usually responsible for binding ECM, whereas β subunit recruits intracellular regulatory proteins^50^. The α subunit including α1, α2, α5, and α11 subunits pairs only with the β1 subunit^29^. Previous study assessing the significance of β subunit in regulating mechanosensitive pathway demonstrates that perturbing β1 integrin signaling in mature osteoblasts causes skeletal abnormalities and loss of adaptation to mechanical loads in mouse model^51^. Another study shows that myoepithelial cells adapt force generation to be optimal at healthy breast tissue stiffness through α5β1 or malignant stiffness via αvβ6^52^. Although the expression of β1 integrin is similar among different SBC groups in the present study (Fig. 6), future investigation the role of β subunits in detection of mechanical stimuli may contribute to a rationale for tissue regeneration.

Taken together, our findings provide unambiguous evidence that spherical SBCs with diameters of 100 and 150 μm enhance osteogenic differentiation of MSCs, and such enhancement is mediated by α2 and α5 integrins. FA and actin cytoskeleton are also involved in the 3D mechanotransduction. The unique scaffold system may help clarify how spherical SBC affects stem cells from the perspective of mechanobiology. Such biophysical cues and their underlying mechanosensitive mechanisms could be taken into consideration in conjunction with biochemical cues for tissue engineering and regenerative medicine.

## Methods

### Experimental design

A three-dimensional (3D) scaffold was fabricated with a homogenous spherical geometry and preciously controlled diameters and porosity levels analogous to the microporous holes in trabecular bone. Cell viability, cell morphology, and osteogenic potential of MSCs in different spherical spatial boundary conditions (SBCs) and on a 2D flat substrate of the same material were evaluated. Furthermore, the relationships between the spherical SBCs and organization of actin cytoskeleton, as well as the distribution of focal adhesion (FA) were investigated. Finally, the spherical SBC-sensitive integrin subtypes in regulating the spherical SBCs-related osteogenic differentiation of MSCs were identified.

### Fabrication of 3D scaffolds with controllable pore sizes

Gelatin was used to generate the porous scaffold as previously reported^53^,^54^. A liquid solution of 7% gelatin (Sigma–Aldrich) was prepared with 1% Pluronic^®^ F127 (Sigma–Aldrich) in sterile deionized (DI) water. Four groups with pore sizes of diameter 50, 100, 150, and 200 μm were generated using a novel microfluidic device by adjusting the liquid flow rates of 30–50 μl/min and gas pressures of 10–30 psi (see Supplementary Fig. S1a). The liquid flow was focused into the air stream flowed-orifices of the devices with diameters of 30, 60, 100, and 150 μm, respectively. The liquid foams were subsequently collected into reservoirs. The liquid foam self-assembled in a crystalline order and was stored at 4 °C followed by immersion in 2.5% glutaraldehyde (Sigma–Aldrich). Next, an open-pore solid foam with an interconnected pore network was achieved by degassing under a vacuum pump (GVD-050A, ULVAC KIKO, Inc., Japan). The generated scaffold was further quenched with 0.5% sodium borohydride (Sigma–Aldrich) in sterile DI water, thereby making it biocompatible with living cells, and then washed with sterile Dulbecco’s phosphate-buffered saline (DPBS; Sigma–Aldrich), yielding a final scaffold measuring 5 mm in diameter and 1 mm in thickness with homogeneous spherical pores for 3D cell culturing (see Supplementary Fig. S1b).

### Culture of MSCs in 3D scaffolds

Commercially available human MSCs (Steminent Biotherapeutics Inc., Taipei, Taiwan) were used. The MSCs were maintained in a medium consisting of Iscove’s Modified Dulbecco’s Medium (IMDM; Sigma–Aldrich) and 10% fetal bovine serum (FBS; Life Technologies, USA) supplemented with 10 ng/ml basic fibroblast growth factor (Sigma–Aldrich) and 1% penicillin-streptomycin-glutamine (PSG; Life Technologies, USA). To avoid cell-cell contact-induced senescence^55^, the cell concentration was fine-tuned so that a single pore in Group I (the minimum pore size) contained a single cell. MSCs were plated at 2,000 cells/cm^2^ on 2D gelatin-coated polystyrene (the flat gel group). A total of 50,000 MSCs were seeded into a scaffold via bibulous filter paper placed under the scaffold.

### Cell viability analysis

Viability of MSCs in 3D scaffolds or on 2D flat gel at 1 day of culturing was determined by live/dead double staining (Molecular Probes, USA) with 2 μM of calcein acetoxymethyl ester and 4 μM of ethidium homodimer-1 for 30 minutes at room temperature (RT) per the manufacturer’s instructions. Live and dead cell images were captured by LSM 700 laser-scanning confocal microscope (Carl Zeiss, Germany) with excitation at 490 nm and 545 nm, respectively. Five random microscopic fields of 0.65 mm × 0.65 mm at 100 × magnification were taken from three individual experiments (*n* = 5 fields). Each condition was independently repeated three times.

### Cell shape analysis

The cell shape of live MSCs in 3D scaffolds or on the 2D flat gel at 1 day of culturing was imaged by inverted microscope (Eclipse Ti-U, Nikon, Japan) and analyzed by ImageJ software (version 1.48, NIH, USA). The aspect ratio (AR) was calculated by the ratio of long- to short-axis in a cell. Five random microscopic fields of 0.5 mm × 0.5 mm at 200 × magnification were taken from three individual experiments. Twenty cells in each group were measured (*n* = 20 cells).

### Scanning electron microscopy (SEM) examination

MSCs in 3D scaffolds at 1 day of culturing were fixed with 2.5% glutaraldehyde (Electron Microscopy Sciences, EMS, USA) for 2 hours, post-fixed with 1% osmium tetroxide (EMS), and rinsed with sterile DPBS followed by washing with DI water. Samples were snap-frozen at –80 °C and lyophilized by a freeze dryer (Gamma 1–20, Christ, Germany). The samples were then mounted on a strip and sputter-coated with gold (Ion Sputter JFC-1200, Jeol, Japan). All images were photographed under an ultra-high resolution SEM (JSM 7600 F, Jeol, Japan) at an accelerating voltage of 5 kV. At least three independent repeats were performed. Cells within a pore in the 3D scaffold were individually pseudocolored using Photoshop CC software (Adobe Systems Incorporated, USA).

### *In vitro* osteogenic differentiation

Induction of differentiation towards the osteogenic lineage was performed using our previously reported protocol^56^. Briefly, each group was treated with an osteogenic medium consisting of IMDM supplemented with 0.1 μM dexamethasone (Sigma–Aldrich), 10 mM β-glycerol phosphate (Sigma–Aldrich), 0.2 mM ascorbic acid (Sigma–Aldrich), and 1% PSG. The medium was changed twice weekly. On 0, 7, 14, and 21 days of induction, cells were collected and gene expression as well as alkaline phosphatase (ALKP) activity were analyzed.

### Mineralization Analysis

Calcium deposition was determined by alizarin red S and von Kossa staining. After 28 days of osteogenic induction, the differentiating MSCs in 3D scaffolds or on 2D flat gel were fixed with 10% formaldehyde (Sigma–Aldrich) and washed with DI water. For alizarin red S staining, samples were incubated with 40 mM alizarin red S (pH = 4.2, Sigma–Aldrich) at RT for 30 min. For von Kossa staining, samples were incubated with 2% silver nitrate (Sigma–Aldrich) at RT in the dark for 10 minutes followed by UV exposure for 45 minutes. After staining, all study groups were washed with DI water and imaged. Each condition was performed independently twice.

### RNA extraction, reverse transcription, and quantitative polymerase chain reaction (qPCR)

Total RNA was extracted from differentiating MSCs using RNeasy Mini Kit (Qiagen, USA) followed by reverse transcription using Moloney murine leukemia virus reverse transcriptase (Promega, USA) according to the manufacturer’s instructions. Transcription levels of target genes were measured by qPCR with TaqMan Fast Universal PCR Master Mix (2X) (Applied Biosystems, USA). Target gene-specific primer sequences and suitable probes were designed by the Universal ProbeLibrary System software and listed in Table 1. The qPCR reaction conditions were one cycle of hot-start activation at 95 °C for 20 seconds, followed by 40 cycles of amplification and detection, including 95 °C for 1 second and 60 °C for 20 seconds using the Roche LightCycler 480 (Roche Applied Science, USA). The relative mRNA expression level of each gene was represented as the ratios of each study group to the 2D flat gel group after normalization to glyceraldehyde 3-phosphate dehydrogenase (GAPDH) as a reference transcript using the ΔΔCT method. At least three independent repeats were performed, each time in duplicate.

### Quantification of ALKP activity

ALKP activity of MSCs in 3D scaffolds or on 2D flat gel was measured using the Alkaline Phosphatase Fluorometric Assay Kit (Abcam, USA) per the manufacturer’s instructions. ALKP activity was normalized to the total amount of genomic DNA using the FluoReporter Blue Fluorometric dsDNA quantitation kit (Molecular Probe, USA) according to the manufacturer’s instructions. At least three independent repeats were performed.

### ALKP staining

After 14 days of osteogenic induction, the differentiating MSCs in the 3D scaffolds or on the 2D flat gel were fixed with 3.7% formaldehyde (Sigma–Aldrich), washed with DPBS, and then incubated with 5-bromo-4-chloro-3-indolyl phosphate/nitro blue tetrazolium (Sigma–Aldrich) in dark at RT for 1 hour. The sample was rinsed in DPBS and imaged by inverted microscope. At least three independent repeats were performed.

### Immunofluorescence staining and image acquisition

MSCs in the 3D scaffolds or on the 2D flat gel at 1 day of culturing were washed with DPBS, fixed in 4% paraformaldehyde (EMS) at RT for 30 minutes, and washed with DPBS, followed by permeabilization with 0.5% Triton X-100 (Sigma–Aldrich) for 10 minutes. After washing with DPBS, the samples were blocked with 2% bovine serum albumin (Sigma–Aldrich) for 1 hour, followed by incubation with primary antibody (1:100, mouse anti-vinculin monoclonal antibody; Sigma–Aldrich or 1:200, rabbit anti-myosin light chain phosphor S20 polyclonal antibody; Abcam) at 4 °C overnight, and then washed with DPBS. After incubation with secondary antibodies (1:100, goat anti-mouse Cy5 conjugated secondary antibody; Jackson ImmunoResearch, USA or 1:200 goat anti-rabbit DyLight 488; Jackson ImmunoResearch, USA) at RT for 1 hour, the samples were washed with DPBS again. Nuclei were stained with DAPI (Sigma–Aldrich). For F-actin, cells were labeled with 6.6 μM rhodamine-conjugated phalloidin (Molecular Probes, USA) for 30 minutes. Images were captured by LSM 700 laser-scanning confocal microscope. At least three independent repeats were performed.

### Image analysis

A quantitative analysis of F-actin, vinculin contents, and vinculin at adhesions was performed as previously described^57^. Because FA formation occurred within a few micrometers at the cell periphery^58^, the immunofluorescence intensities of vinculin at adhesions within 5 μm of the spatial boundary were quantified. The z-stacks images of MSCs in 3D scaffolds were captured using a 20 × /0.70 dry objective at a pixel resolution of 512 × 512 with a z-step size of 1 μm and an average of 2 frames. Laser power, gain, and offset were kept constant across the study groups. The immunofluorescence intensity of a single cell was calculated by measuring the total pixel values using MetaMorph software (Molecular Devices, USA). FA area was analyzed by ImageJ software. At least ten cells were calculated in each condition from three independent experiments.

### siRNA transfection

The siRNA targeting α2 and α5 integrins were purchased from Invitrogen and transfected into MSCs according to the manufacturer’s instructions. The sequences are listed in Supplementary Table S2. The experimental flow chart of siRNA knockdown is described in Supplementary Fig. S7.

### Statistical analysis

All statistical analyses were carried out using SPSS software (version 19.0, IBM, USA). Student’s *t*-test was used for two group analysis with significant differences indicated by asterisks (*p < 0.05, **p < 0.01, ***p < 0.001) to compare the siRNA experiments. One-way analysis of variance (ANOVA) followed by Tukey’s post-hoc tests were performed for multiple comparisons. A *p*-value less than 0.05 was defined as being statistically significant. Groups with different letters are significantly different from one another while those with the same letter are not.

## Additional Information

**How to cite this article**: Lo, Y.-P. *et al.* Three-dimensional spherical spatial boundary conditions differentially regulate osteogenic differentiation of mesenchymal stromal cells. *Sci. Rep.* **6**, 21253; doi: 10.1038/srep21253 (2016).

## Supplementary Material

Supplementary Information

## Figures and Tables

**Table 1:** Primer sequences and probes from Universal ProbeLibrary used in qPCR analysis.

Gene name	Primer sequences	Probe number
Runx2	5′-gtgcctaggcgcatttca-3′	29
	5′-gctcttcttactgagagtggaagg-3′	
Osterix	5′-taacctgatggggtcatggt-3′	43
	5′-gactgcagagcaggttcctc-3′	
ColI A1	5′-cccctggaaagaatggagat-3′	60
	5′-aatcctcgagcaccctgag-3′	
Osteonectin	5′-gtgcagaggaaaccgaagag-3′	77
	5′-tgtttgcagtggtggttctg-3′	
Vinculin	5′-gatgaagctcgcaaatggtc-3′	28
	5′-tctgcctcagctacaacacct-3′	
α1 integrin	5′-aattggctctagtcaccattgtt-3′	14
	5′-caaatgaagctgctgactggt-3′	
α2 integrin	5′-tcgtgcacagttttgaagatg-3′	7
	5′-tggaacacttcctgttgttacc-3′	
α5 integrin	5′-cccattgaatttgacagcaa-3′	55
	5′-tgcaaggacttgtactccaca-3′	
α11 integrin	5′-gaggctgacgtcctcttcac-3′	66
	5′-gttgggcttgacctcgtagt-3′	
β1 integrin	5′-cgatgccatcatgcaagt-3′	65
	5′-acaccagcagccgtgtaac-3′	
GAPDH	5′-agccacatcgctcagacac-3′	60
	5′-gcccaatacgaccaaatcc-3′	

Abbreviation: Runx2, runt-related transcription factor 2; ColI A1, Type I collagen alpha1; GAPDH, glyceraldehyde-3-phosphate dehydrogenase.
