# Supplementary material for: Three-dimensional spherical spatial boundary conditions differentially regulate osteogenic differentiation of mesenchymal stromal cells
Source: Sci Rep. 2016 Feb 17;6:21253. doi: 10.1038/srep21253 (PMC4756701; doi:10.1038/srep21253)
Supplement: Supplementary Information [file srep21253-s1.doc]

**Supplementary Information for:**

**Three-dimensional spherical spatial boundary conditions differentially regulate osteogenic differentiation of mesenchymal stromal cells**

Yin-Ping Lo^a^, Yi-Shiuan Liu^b^, Marilyn G. Rimando^c^, Jennifer Hui‐Chun Ho^d, e, f^, Keng-hui Lin^g^, and Oscar K. Lee^h, i, j, *^

**Supplementary Figure S1.**

**
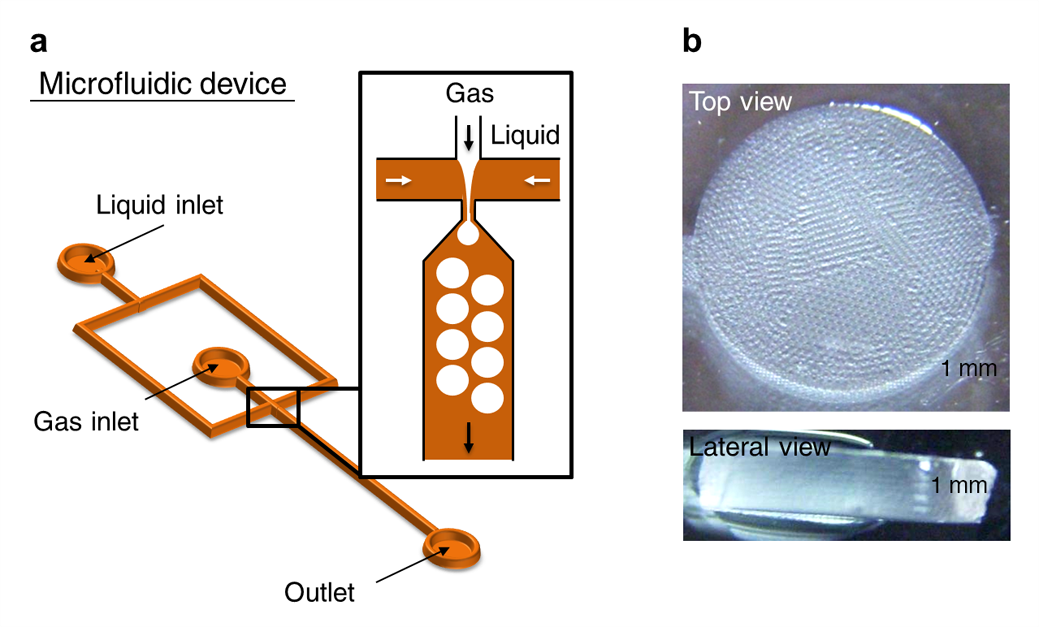
**

**Supplementary Figure S1. Fabrication of the 3D scaffold with uniform-sized pores.**

(**a**) Schematic diagram of polydimethylsiloxane flow-focusing microfluidic device. The magnified box illustrated the junction of uniform bubble generation. (**b**) Disc-shaped porous scaffold with a diameter of 5 mm and a thickness of 1 mm. (Supplementary Fig. S1a drawn by Yin-Ping Lo).

**Supplementary Figure S2.**

**
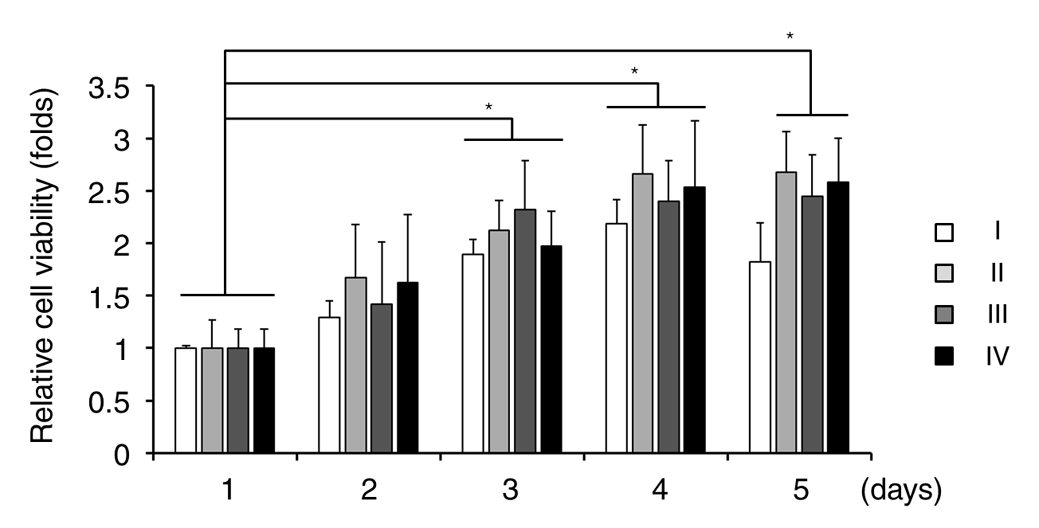
**

**Supplementary Figure S2. Cell viability of MSCs in the 3D scaffolds (Groups I, II, III, and IV) at different time points.**

Cell viability of MSCs in the 3D scaffolds at 1, 2, 3, 4, and 5 days of culturing in the maintenance medium. Data were represented as mean ± SD of the ratios of each study group to that of day 1, *n* = 3. Groups with different time points were compared to that of day 1. Significant difference (Student’s *t*-test; **p* < 0.05) was indicated by asterisks.

**Supplementary Figure S3.**

**
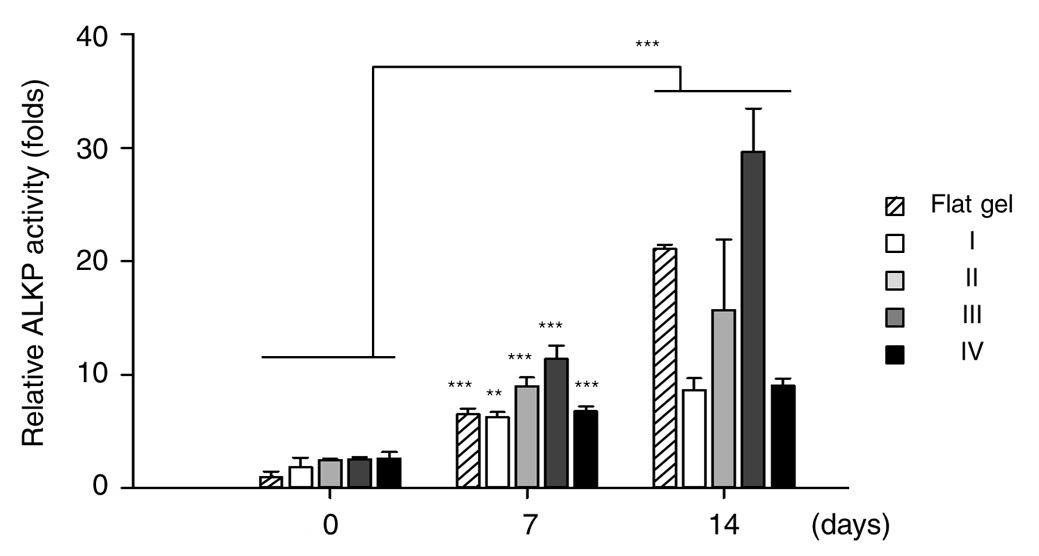
**

**Supplementary Figure S3. ALKP activity of differentiating MSCs in the 3D scaffolds (Groups I, II, III, and IV) and on the 2D flat gel.**

Analysis and quantification of ALKP activity of differentiated MSCs in the 3D scaffolds or on the 2D flat gel at 0, 7, and 14 days of culturing in the osteogenic medium. Data were represented as mean ± SD of the ratios of each study group to the flat gel group at d0, *n* = 3. Groups with induction were compared to those without. Significant difference (Student’s *t*-test; ***p* < 0.01, ****p* < 0.001) was indicated by asterisks.

**Supplementary Figure S4.**


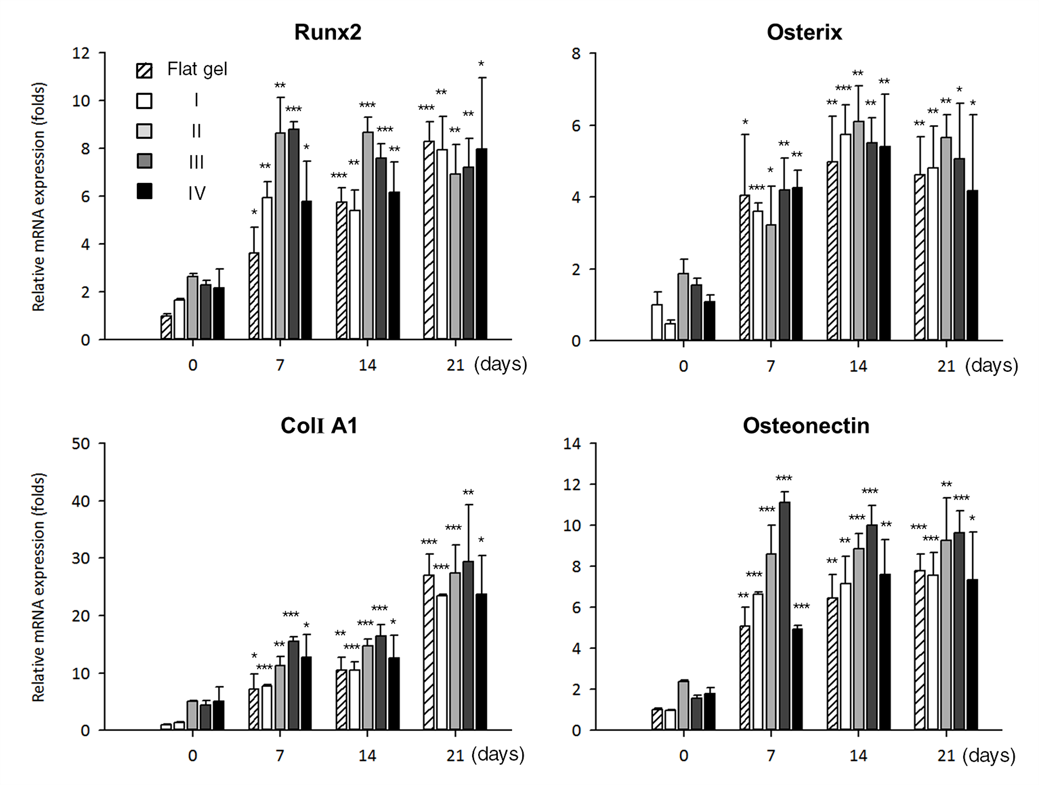


**Supplementary Figure S4. Osteoblast-related genes expressions of differentiating MSCs in the 3D scaffolds (Groups I, II, III, and IV) and on the 2D flat gel.**

Osteoblast-related gene expressions of differentiated MSCs in the 3D scaffolds or on the 2D flat gel were determined by qPCR at 0, 7, 14, and 21 days of culturing in the osteogenic medium. Data were represented as mean ± SD of the ratios of each study group to the flat gel group at d0, *n* = 3. Groups with induction were compared to those without. Significant difference (Student’s *t*-test; **p* < 0.05, ***p* < 0.01, ****p* < 0.001) was indicated by asterisks.

**Supplementary Figure S5.**


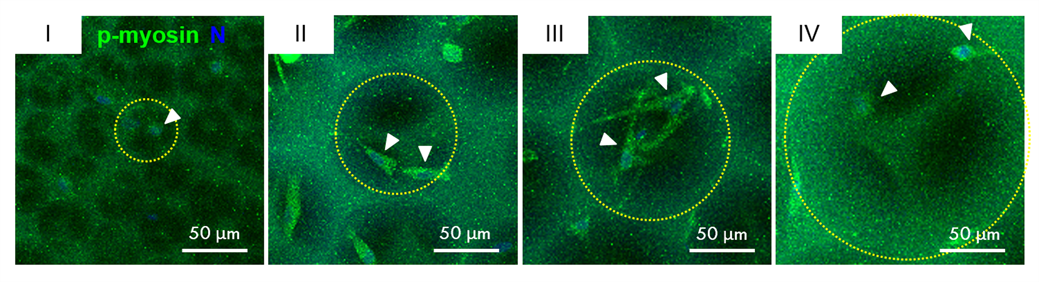


**Supplementary Figure S5. Active phosphorylated myosin light chain of MSCs in the 3D scaffolds (Groups I, II, III, and IV).**

Fluorescent color maps of z-projection resulting from immunostaining demonstrated the active phosphorylated myosin light chain of MSCs in the 3D scaffolds. The cells with p-myosin were indicated by arrowheads. The spatial boundaries were marked by yellow dotted line. Green, p-myosin; Blue, nucleus.

**Supplementary Figure S6.**


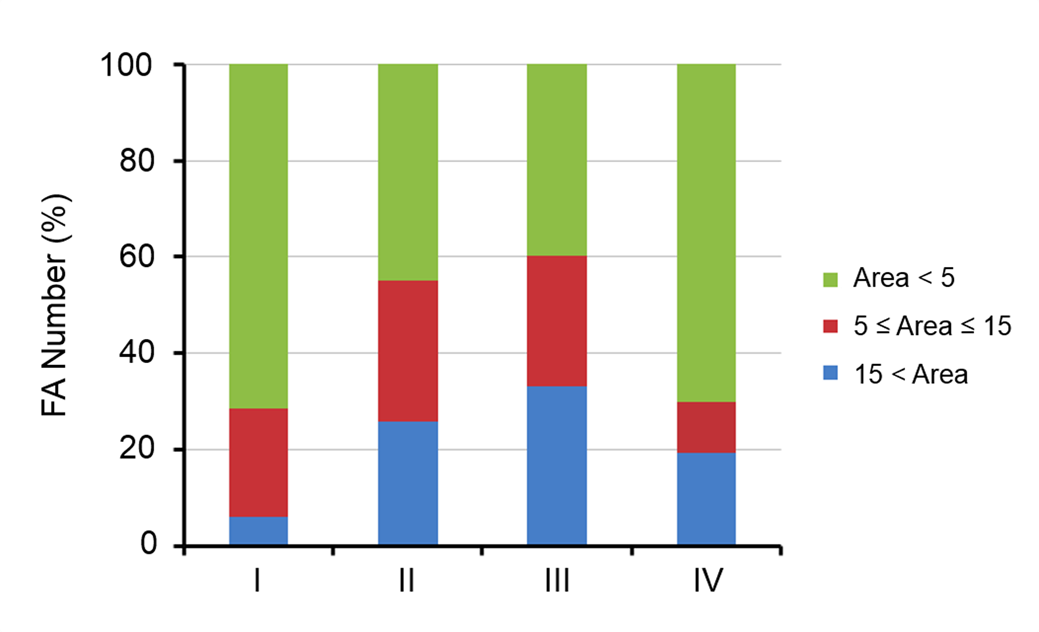


**Supplementary Figure S6. Quantification of FA size from MSCs in the 3D scaffolds (Groups I, II, III, and IV).**

Analysis and quantification of FA area at the cell extremities by ImageJ software. At least ten cells were calculated in each condition from three independent experiments. The unit of area, μm^2^.

**Supplementary Figure S7.**


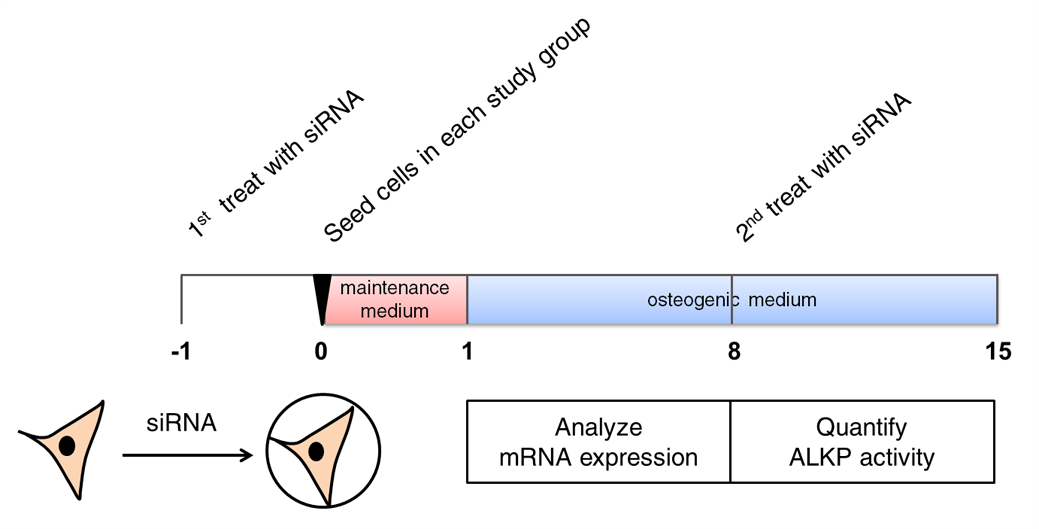


**Supplementary Figure S7. Experimental flow chart of siRNA knockdown study design.**

MSCs were cultured, treated with 10nM siRNA at 37^o^C for 6 hours, and kept in the maintenance medium for 24 hours before being re-seeded in the 3D scaffolds or re-plated on the 2D flat gel. The 2^nd^ siRNA transfection was performed at 7 days of culturing in the osteogenic medium. MSCs treated with scrambled siRNA served as controls. Knockdown efficiency, transcription levels of osteoblast-related markers, and ALKP activity were further determined by qPCR and quantitative ALKP activity. (Supplementary Fig. S4 drawn by Yin-Ping Lo).

**Supplementary Table S1. 3D structural geometrical properties of the fabricated scaffolds.**

| **Group** | **I** | **II** | **III** | **IV** |
| --- | --- | --- | --- | --- |
| Pore diameter, d (μm) | 47.0 ± 2.2 | 84.8 ± 11.0 | 147.9 ± 7.2 | 198.7 ± 9.1 |
| *Pore radius, r = 1/2 d | 1 | 2 | 3 | 4 |
| *Pore surface curvature = 1/r | 12 | 6 | 4 | 3 |
| *Pore volume, V = 4/3 πr^3^ | 1 | 2^3^ | 3^3^ | 4^3^ |
| Scaffold storage modulus (KPa) | 3.3 ± 0.6 | 3.4 ± 0.8 | 4.1 ± 0.5 | 3.6 ± 0.5 |

Asterisk (*) denotes the properties represented as a relative ratio among each group. Note that the storage moduli were not statistically different between these four groups (*p* > 0.22). Data were represented as mean ± S.D., *n* = 5.

**Supplementary Table S2. siRNA sequences from Invitrogen used in knockdown analysis.**

| **Gene name** | **siRNA sequences** |
| --- | --- |
| α2 integrin | 5’- gccugcagaagaauaugguaguaaa -3’  5’- uuuacuaccauauucuucugcaggc -3’ |
| α5 integrin | 5’- ccgaguaccugaucaaccugguuca -3’  5’- ugaaccagguugaucagguacucgg -3’ |

**Supplementary Methods.**

**Mechanical measurement of the 3D scaffolds**

Mechanical properties of the fabricated scaffolds were characterized using the shear storage modulus to represent the elastic characteristic of the viscoelastic material in response to the applied force. Measurements were done with a rotational rheometer (Physica MCR 301, Anton Paar, Austria). Individual samples were held by a parallel plate holder (D-CP/PP7, Anton Paar, Austria) and subjected to oscillatory shear with a strain amplitude of 0.005% at a constant frequency of 1 Hz. Five scaffolds were measured in each group and fabricated from three independent experiments. Each scaffold was performed in triplicate (*n* = 5 scaffolds). Each condition was repeated three times independently, each time in duplicate.

**MTS assay**

Viability of MSCs in the 3D scaffolds at 1, 2, 3, 4, and 5 days of culturing was determined by MTS assay (Promega, USA) according to the manufacturer’s instructions. Briefly, 100 μl pre-warmed basal medium supplemented with 20 μl MTS solution was added into each sample. Samples were incubated in the dark at 37^o^C for 1 hour. The absorbance of the formazan product at an optical density (OD) of 490 nm was then measured using a microplate reader (VERSAmax, Molecular Devices, USA). At least three independent repeats were performed in each condition.
